# Systematic Review of Interventions Addressing Food Insecurity in Pregnant Women and New Mothers

**DOI:** 10.1007/s13668-022-00418-z

**Published:** 2022-05-02

**Authors:** Fiona H. McKay, Sheree Spiteri, Julia Zinga, Kineta Sulemani, Samantha E. Jacobs, Nithi Ranjan, Lauren Ralph, Eliza Raeburn, Sophie Threlfall, Midina L. Bergmeier, Paige van der Pligt

**Affiliations:** 1 School of Health and Social Development/Institute for Health Transformation, Faculty of Health, Deakin University, Victoria, Australia; 2 Institute for Physical Activity and Nutrition (IPAN), School of Exercise and Nutrition Sciences, Deakin University, Geelong, Australia; 3 Department of Nutrition and Dietetics, Western Health, Footscray, Australia

**Keywords:** Food security, Pregnancy, Intervention, Review

## Abstract

***Purpose of the Review*:**

Food insecurity can have a negative health impact for women during pregnancy and the postpartum period; however, there are a range of barriers to meeting nutritional guidelines during pregnancy. Food insecurity is associated with an increased risk of pregnancy complications and mental and physical health outcomes. This review aims to provide insight into programmes and interventions which have targeted food insecurity in pregnant and early postpartum women. The central research question for this review is as follows: What programmes and interventions have sought to address food insecurity among pregnant and postpartum women? A systematic search of five electronic databases including Medline, CINAHL, Global Health, Embase, and Cochrane was undertaken on August 2021. Key thematic areas searched were food insecurity, pregnancy, nutritional outcomes, and interventions or programmes. Only studies that were published since 2000 in English were considered.

***Recent Findings*:**

Eleven studies were included in this review. Studies employed a range of methods and outcomes measures. They were conducted in mostly low- and middle-income countries, and in general, focused on nutritional supplementation, with some studies also incorporating nutrition education or counselling.

***Summary*:**

The findings of this review suggest that while there are a range of possible interventions that seek to address food insecurity and hunger among pregnant and postpartum women, the limited number of robust evaluations or long-term interventions mean that evidence for any one intervention type is limited. Furthermore, the programmes and interventions that do exist are generally embedded within a single context or structure, and as such, may not be able to be widely implemented. (*Prospero Registration* CRD42022245787)

## Introduction

Food security exists when people have physical, social, and economic access to sufficient, safe, and nutritious food to meet their dietary needs and food preferences for a healthy life [1]. Food insecurity, the absence of safe and secure nutritious food, both with or without hunger, is a major public health issue in both high income and low- and middle-income countries, with an estimated 2 billion people globally considered to be food insecure [2]. While food insecurity is present across all regions, countries within Africa tend to have the lowest levels of food security, with the Food and Agriculture Organisation (FAO) [1] suggesting that almost 60% of people in African nations were food insecure in 2020, compared to 25% in Asian nations, 40% in Latin America and the Caribbean, and 8.8% in North America and Europe.

Food insecurity can have a negative impact on a range of health outcomes which are more pronounced in some population groups. Pregnant and postpartum women are especially vulnerable to the negative impacts of food insecurity, as they have increased nutritional requirements during pregnancy to meet the needs of the growing foetus [3], while after pregnancy they may have additional nutritional requirements for breastfeeding [4]. Research suggests that the dietary intakes of pregnant women often do not meet nutritional guidelines [5•], and while some studies have explored the role of nutritional counselling [6], pregnant women may need more assistance in meeting their nutritional needs at this time.

Pregnancy can be a physically and mentally demanding time where food insecurity can exacerbate stress [3]. Many studies have found an association with food insecurity, and reduced quality of life and psychosocial outcomes such as increased depression and anxiety [7, 8]. Furthermore, food insecurity can be associated with an increased risk of pregnancy complications including gestational diabetes, anaemia, and pregnancy-induced hypertension [9]. Food insecurity is also associated with poor health outcomes for the baby including low birth weight, increased risk of birth defects [10], and poor developmental outcomes [8, 9, 11, 12].

Given the serious and wide-reaching implications of food insecurity, finding solutions to food insecurity and hunger are becoming increasingly urgent. However, the most effective responses to hunger and food insecurity remain unknown. Traditional responses include emergency and community food aid and supplemental food programmes; however, these responses are increasingly being recognised as unable to meet the needs of those who are food insecure and hungry [13]. This review aims to provide insight into the programmes and interventions conducted to-date which have targeted food insecurity in pregnant and early postpartum women. The central research question for this review is as follows: *What programmes and interventions have sought to address food insecurity among pregnant and postpartum women?* The findings of this review can be used to inform government departments, non-government practitioner organisations, and public health bodies regarding the effectiveness of a range of potential interventions aimed at addressing food insecurity and hunger among pregnant and postpartum women.

## Method

A systematic search of five electronic databases, Medline, CINAHL, Global Health, Embase, and Cochrane, was undertaken on August 2021. Key thematic areas searched were food insecurity, pregnancy, nutritional outcomes, and interventions or programmes, see Table 1 for details of search terms. To gain a comprehensive collection of recently published articles that report on interventions to address food insecurity among pregnant and postpartum women, only studies that were published since 2000 were considered. In addition, only English language peer-reviewed articles were considered.

**Table 1:** Key search terms used in academic literature search

Food insecurity	Pregnancy	Nutritional interventions
“food insecur*” OR “food access*” OR “food afford*” OR “food poverty*” OR “food secur*” OR “food suppl” OR “food sufficien*” OR “food insufficien*” OR “food desert*” OR (hunger OR hungry) OR “food Assist*” OR “Food shortage”	pregnan* OR gestation* OR maternal OR antenatal OR postnatal OR “post natal” OR postpartum OR “post partum” OR childbirth OR “child birth” OR Prenatal OR “Pre natal” OR “Recently Delivered” OR mother* OR father* OR parent*	intervention*OR strateg* OR program* OR activit* OR (policy OR policies) OR implement* OR guideline* OR Education OR “Nutrition intervention” OR “Nutrition programmes” OR “Nutrition program*”

^*^Truncation used at the end of the word in all databases to retrieve all suffix variation

**Fig. 1:**
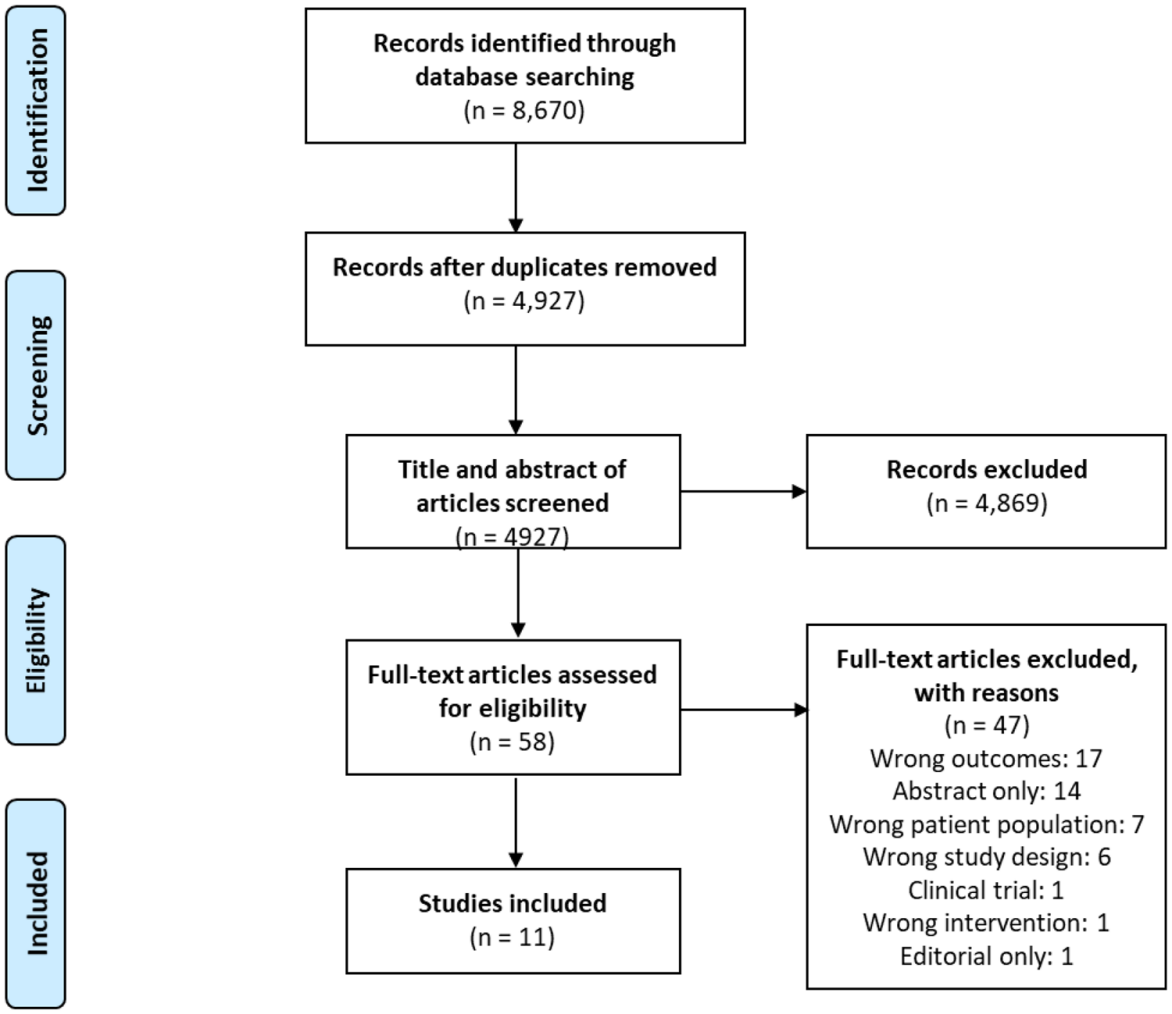
Prisma flow diagram of systematic search

Two authors independently reviewed all articles to identify relevant studies. Articles underwent a three-step selection process (see Fig. 1). Articles were imported into Covidence, a web-based systematic review management package [14]; duplicates were identified and removed. Articles were first screened by title and abstract based on the inclusion and exclusion criteria outlined above. Any article that clearly did not meet the inclusion criteria was removed at this stage, any that did or possibly could meet the inclusion criteria were retained. Full text of the remaining articles were obtained for further assessment. At least two authors independently read all remaining articles to determine whether the article met the inclusion criteria. Any articles at this stage that clearly did not meet the inclusion criteria were removed, and disagreements were discussed and settled by consensus between authors.

### Data Extraction and Analysis

Data were extracted by all authors. Data including key characteristics of the study or report, research and data collection method, outcomes, and intervention if available were extracted into a table for analysis. Given the variety of data present, data were also thematically analysed following the constant comparative method [15]. This allowed reviewers to draw common themes from the data. Thematic analysis considered the main themes identified within each of the included papers and then consisted of a comparison of these themes across each of the papers resulting in a discussion of the main thematic areas across all papers included.

This review adheres to the PRISMA Statement [16, 17] and has been registered with the international prospective register of systematic reviews (PROSPERO: CRD42022245787).

### Quality Assessment

Studies were assessed for quality and risk of bias according to the Academy of Nutrition and Dietetics Evidence Analysis Library, Quality Criteria Checklist [18]. Four key questions regarding relevance address the practice applicability of the study, with scientific soundness analysed through 10 key validity questions that encompass issues of inclusion/exclusion, bias, generalisability, and data collection and analysis. A rating of positive, negative, or neutral is allocated to a study based on the answers to the 10 validity questions. A study was deemed positive if most of the validity question answers were “Yes,” including affirmative assessment of four essential criteria relating to subject selection, comparable groups, intervention description, and valid measurement of outcomes. A study was rated neutral if any of the four essential validity questions regarding subject selection, comparable groups, intervention description and valid measurement of outcomes are answered “No,” but other areas indicate strengths. A negative rating was given if most of the answers to the ten validity questions were “No.” Two authors independently assessed study quality and any discrepancies were resolved through discussion.

## Results

The search generated 8670 articles, of which 3743 were duplicates. The titles and abstracts of 4927 articles were read; 4869 articles were excluded because they did not refer to or measure either directly or indirectly, food insecurity among pregnant or postpartum women, leaving 58 articles for full text review. The full text of 58 articles was reviewed; 47 articles were excluded as they did not meet the inclusion criteria. The remaining 11 studies were included in this review (Table 2).

**Table 2:** Summary of studies included in review

**Reference**	**Location**	**Study aim**	**Study population**	**Participant characteristics**	**Intervention**			**Food security measures**	**Outcomes**
					**Study design**	**Type**	**Components**		
Briaux et al. [19•]	Togo	Improve the health and nutrition of mother–child pairs through cash transfer	1357 women who were at least 3 months pregnant and mothers of children aged 0–23 months	No significant differences between control and intervention arms at baseline in any household sociodemographic and economic characteristics	Nonblinded parallel-cluster–randomised controlled trial	Cash transfer programme	162 villages randomised into either a control arm (community case management of childhood illnesses and acute malnutrition programme and other activities) or intervention arm (intense activities)	Household Food Insecurity Access Score (HFIAS)	Height for weight and stunting. Cash transfer had positive primary outcomes. Cash transfer positively impacted mothers’ and children consumption of animal source foods and household food insecurity, but no impact on reported child morbidity 2 weeks prior to report but did reduce financial barriers to healthcare
Frith et al. [8]	Bangladesh	Early invitation for prenatal food supplementation programme in reducing the negative influence of food insecurity on maternal-infant interaction	A cohort of 180 mother-infant dyads	No differences at baseline for food supplementation. Average age 26 years, parity 1.5, education 6–7 years	Cohort study. Women randomly assigned start time for receiving food supplement (early ~ 9 weeks or at the usual start time ~ 20 weeks gestation)	Food supplementation	Pregnant women received and consumed supplied food supplements	11-item, experienced-based measure including availability, access, and perceptions of food insecurity	Early invitation time to start a prenatal food supplementation programme resulted in severely food-insecure mother-infant dyads exhibiting similar quality of maternal-infant interaction as more food dyads
Frongillo et al. [29•]	Bangladesh	To determine if participation in nutrition-focused antenatal care would reduce household food insecurity	2000 women with children aged < 6 mo and 600 pregnant women in the 2nd and 3rd trimester	Both groups similar maternal and household characteristics at baseline. Mean age of women 24 y, and most not working outside the home, average 6 y education	Cluster-randomised, nonblinded	Education	The intervention package, included components to reduce food insecurity, delivered through antenatal care and interpersonal communication, community mobilisation, and monitoring weight	Household Food Insecurity Access Scale (HFIAS)	Household food insecurity was reduced in areas where the nutrition-focused antenatal care and community mobilisation intervention package was implemented
Heberlein et al. [21]	USA	Compare the effects of group to individual antenatal care in late pregnancy and early postpartum on women’s food security and psychosocial outcomes	248 diverse, low-income pregnant women with low obstetric risk	Both groups similar age and education, more black women in the intervention group, while more women in the control group were pregnant with their first child	Prospective cohort study to compare group vs individual prenatal care service delivery	Education	Group care participants attended 10 × 2-h education sessions. Sessions included nutrition, exercise, relaxation techniques, pregnancy symptoms and comfort measures, infant care and breastfeeding, communication, self-esteem, abuse issues, parenting, and prep for childbirth	Household Food Security Survey Module-Short Form (HFSSM)	Group participants more likely to become food-secure postpartum and to remain food-secure postpartum. Group care participants had higher maternal-infant attachment scores than intervention care in early pregnancy survey
Leroy et al. [22]	Burundi	To assess the impact of the “Tubaramure” programme on household food consumption and security, maternal dietary diversity, and infant and young child feeding practices; the role of the food rations; and the impacts on children after the programme	2598 pregnant women mothers of children aged < 6 months	Household head average age 34-35yrs, 38–42% no formal education; Mothers average age 28-29yrs, 49–54% no formal education	4-arm cluster-randomised controlled repeated cross-sectional design	Food supplementation	3 components (1) monthly household food ration for pregnant women daily until offspring was 6 months; (2) improved health services; (3) twice monthly education	Household Food Insecurity Access Score (HFIAS) and Household Hunger Score (HHS)	Proportion of food-secure households higher in the treatment compared to control arms. Impact on household energy consumption similar across treatment arms and positive effect on maternal dietary diversity and children consuming > 4 food groups
Metallinos et al. [23]	USA	The association between the duration of WIC participation and household food security	79,240 nulliparous pregnant women eligible for the WIC programme	Maternal Age: 22.6yrs, Race: 16.5% Black, 28.5% Hispanic, 47.9% White, 7.1% Asian, Trimester of WIC entry: 48.5% 1st tri, 40.4% 2nd tri, 11.1% 3rd tri	Longitudinal study	Food supplementation	The Special Supplemental Nutrition Programme for Women, Infants and Children (WIC) allows for supplemental foods, health care referrals and nutrition education	4 question subscale of Household Food Security Survey Module (HFSSM)	Earlier and longer WIC participation associated with improved household food security status. An additional WIC visit reduced the odds of any household food insecurity
Mridha et al. [24]	Bangladesh	The provision of lipid-based nutrition supplements to pregnant and lactating women would result in positive nutritional change	4011 pregnant and post-partum women (6 months)	1047 in the experimental group and 2964 in the 3 control groups	Cluster RCT	Food supplementation	48 clusters received iron and folic acid and 16 clusters received lipid-based nutrition supplements	Household Food Insecurity Access Score (HFIAS)	Infants in the experimental group had higher weights and were less likely to be stunted. Household food security not reported
Phojanakong et al. [25]	USA	The effectiveness of a trauma-informed intervention to reduce household food insecurity	372 parents of children aged < 6 years, participating in Temporary Assistance for Needy Families and SNAP	Mean age 28 years, 94.1% female, 91.1% Black, 75% high school or more	Single-arm cohort intervention	Counselling	16 sessions of trauma-informed programming incorporated healing-centred approaches to address previous exposures to trauma	Household Food Security Survey Module (HFSSM)	Full participation had 55% lower odds of facing HFI compared with the low/no participation group
Raghunathan et al. [26]	India	The effect of conditional cash transfers with the Mamata scheme on the delivery and uptake of nutrition interventions and household food security	1161 pregnant women and/or mothers with up to two live births with children aged 0–24 months	Age: > 19 years, 25.8 no education 20.4%, primary school 17.1%, socioeconomic status: wealth SES quintile: 1 (low) 12.1% — 5 (highest) 26.6%	Cohort study	Cash transfer programme	The Mamata scheme provided a partial wage compensation to pregnant and lactating mothers Direct payments were made in four instalments payable at the end of the second trimester, and at 3, 6, and 9 months after delivery, conditional on antenatal check-up, vaccination, counselling sessions and exclusive breastfeeding	Household Food Insecurity Access Score (HFIAS)	cash transfers were associated with decrease in the overall HFIAS score. Purchasing from the Public Distribution System was associated with a larger decrease in the overall food insecurity. Cash transfer was associated with increased household savings and expenditure on food, expenditure on child health, food and care, expenditure on maternal health, care, and nutrition
Rifayanto et al. [28]	Indonesia	Effectiveness of nutrition education on knowledge and attitudes and the impact of egg and milk supplementation on nutritional status of pregnant women	45 pregnant women in their 2nd and 3rd trimester	18–40 years old	Cohort pre-experimental study design (one group pre-test post-test)	Education and supplementation	Nutrition education intervention three meetings with pregnant women. Nutrition education was provided through lectures and discussions. Egg and milk supplementation for pregnant women is given every day for 90 days	Household Food Insecurity Access Scale (HFIAS)	Consumption of protein, vegetables and fruit increased. Nutrition knowledge after nutrition education increased. Additional food in the form of egg and milk for 90 days was effective in increasing the mid-upper arm circumference. 44% of households achieved food security
Sibson et al. [27]	Niger	To test if starting the cash transfer 2 months earlier, (same amount of cash) would reduce the prevalence of acute malnutrition in children	2073 pregnant and lactating women and children, aged 6–59 months	Children, aged 6–59 months living in beneficiary households	Cluster‐randomised controlled trial	Cash transfer programme	Cash transfer intervention for 4 months. Cash given to female household representatives to be used to purchase a food basket. Beneficiaries required to attend education session, women and children were screened for acute malnutrition	Household Food Insecurity Access Score (HFIAS)	Starting the cash transfer earlier and providing the same amount of cash over 6 months instead of 4, alongside 4 months supplementary feeding, temporarily increased beneficiary food security, but did not impact on children’s nutritional status at end line

Based on the quality assessment and risk of bias analysis (Table 3), six papers were considered positive quality studies that adequately addressed the majority of the ten validity questions, including the four essential criteria [8, 19•, 20–23]. Four papers received a neutral rating due to inadequately fulfilling essential criteria regarding either subject selection, comparable groups, intervention description, or valid measurement of outcome [24–27]. One study received a negative rating due to inadequately addressing eight of the ten validity criteria [28].

**Table 3:** Assessment of study quality

**Study**	**Research statement**	**Selection bias**	**Comparable study groups**	**Withdrawals**	**Blinding**	**Intervention**	**Outcomes measured**	**Statistical analysis**	**Conclusions supported by results**	**Bias unlikely**	**Overall quality rating**
Briaux et al. [19•]	Yes	Yes	Yes	No	No	Yes	Yes	Yes	Yes	Yes	+
Frith et al. [8]	Yes	Yes	Yes	Yes	No	Yes	Yes	Yes	Yes	Yes	+
Frongillo et al. [29•]	Yes	Yes	Yes	No	N	Yes	Yes	Yes	Yes	Yes	+
Heberlein et al. [21]	Yes	Yes	Yes	Yes	No	Yes	Yes	Yes	Yes	Yes	+
Leroy et al. [22]	Yes	Yes	Yes	Yes	No	Yes	Yes	Yes	Yes	Yes	+
Metallinos-Katsaras et al. [23]	Yes	Yes	Yes	NR	NR	Yes	Yes	Yes	Yes	Yes	+
Mridha et al. [24]	Yes	Yes	Yes	Yes	Yes	Yes	Unclear	Yes	No	Unclear	Ø
Phojanakong et al. [25]	Yes	Yes	N/A	Yes	N/A	Yes	Yes	Yes	Yes	Yes	Ø
Raghunathan et al. [26]	Yes	Unclear	No	N/A	N/A	N/A	Yes	Yes	Unclear	Yes	Ø
Rifayanto et al. [28]	Yes	Unclear	No	No	No	No	No	No	No	Yes	-
Sibson et al. [27]	Yes	Yes	Yes	Yes	No	Yes	Unclear	Yes	Yes	Yes	Ø

Quality of evidence determined by using the Academy of Nutrition and Dietetics Evidence Analysis Library (EAL) Quality Criteria Checklist for Primary Research; NR, not reported; no (weak); unclear (moderate); yes (strong) rating for each component; overall ratings +  = positive, Ø = neutral and—= negative

A summary of the included studies and data extraction is presented in Table 2. Study designs of the interventions varied, five were randomised controlled trials (RCT) [19•, 22, 24, 27, 29•], five studies were cohort studies [8, 21, 25, 26, 28], and one was a longitudinal study [23]. Studies were conducted in seven countries: three each in Bangladesh [8, 24, 29•] and the USA [21, 23, 25], and one each in Burundi [22], India [26], Indonesia [28], Niger [27], and Togo [19•]. Participant numbers ranged in size from the smallest study with 45 participants [28] to large population level studies with close to 80,000 participants [23].

### Interventions

Articles included in this review reported on a range of interventions. Four articles reported on food or nutrient supplementation [8, 22–24]. Frith and colleagues [8] investigated the impact of a prenatal food supplementation programme earlier in pregnancy, on the relationship between food insecurity and the maternal-infant interaction. Findings of this intervention suggest the earlier in pregnancy a food supplementation intervention can occur, the better for maternal-infant interaction for mothers who are food insecure. Metallinos-Katsaras and colleagues [23] examined association between duration of engagement with The Special Supplemental Nutrition Programme for Women, Infants, and Children (WIC) and the status of household food security. Findings of this intervention suggest that earlier and longer engagement with the WIC programme increase the likelihood that households will be food secure. Leroy et al. [22] evaluated the effectiveness of a programme that delivered food rations along with an integrated health and nutrition education programme (*Tubaramure*) on household food insecurity. This programme, compared to a control group, was found to significantly (*p* < 0.05) improve the proportion of food secure households and increase consumption of both energy and nutrients in study households, and it was also found to have a positive impact on maternal diet diversity. Mridha and Matias [24] explored the impact of lipid based nutritional supplementation during pregnancy on birth outcomes including weight and length, finding that compared to women in the control group, women in the study group had better birth outcomes.

Three studies explored the role of nutritional education in improving food insecurity. Heberlein et al. [21] explored the impact of direct engagement with pregnant women through onsite group and individual prenatal care on food security and psychosocial wellbeing. This study found that group prenatal care, compared to routine, individual care, led to improved food security in pregnant women and better maternal infant attachment, suggesting that such group interactions provide an opportunity to share resources and knowledge. Frongillo and Nguyen [29•] explored if participation in nutrition-focused antenatal care education about improving household knowledge of nutrition could reduce household food insecurity. The findings of this study suggest that participation in nutrition-focused education could lead to improved household food security among pregnant and recently delivered women. Rifayanto et at. [28] explored the role of both nutritional supplementation and an education programme on nutritional knowledge. Findings of this study suggest that nutrition education led to increasing nutritional knowledge among pregnant women, and supplementation of egg and milk for 3 months increased mothers’ mid-upper arm circumference, an assessment of acute malnutrition. However, this study did not have the power to delineate between the impact of the educational programme or supplement on the knowledge and nutritional status of pregnant women.

Three studies explored the impact of cash transfers on maternal outcomes and food insecurity. Raghunathan [26] explored the impact of a cash transfer scheme on a variety of maternal outcomes including receipt of antenatal care and micronutrients, receipt of counselling on infant feeding, breastfeeding, and household food insecurity in a state in India. Findings of this study suggest that this cash transfer programme resulted in improved maternal outcomes and decreased household food insecurity. In a study exploring the impact of a cash transfer programme on child health, mother to child health, and household food insecurity in Togo, Briaux et al. [19•] found that the cash transfer programme lead to improvements in health for both the child and mother and to increase household food security. Sibson et al. [27] explored the impact of additional cash transfers and supplemental feeding programmes on maternal and child health in Niger. While this study found that overall participants reported improved food access, it was not significant when compared with the control group, suggesting that non-food drivers of food insecurity, such as disease, will impede the impact of cash transfers alone.

The final intervention study was that of Phojanakong et al. [25] who explored the role of trauma-informed programming on household food insecurity for people in receipt of government food supplementation programmes. Participants engaged in a weekly counselling session, with results suggesting that increased engagement and attendance with the trauma-informed programming can have an impact on household food insecurity for families with young children by mitigating and treating underlying depression.

### Food Insecurity: Measures and Impact

All interventions included in this review sought to address food insecurity as a main outcome. The studies employed a variety of tools in measuring and assessing food insecurity. The most frequently used measurement tool was the Household Food Insecurity Access Score (HFIAS); this tool was used in seven studies [19•, 22, 24, 26–28, 29•]. The HFIAS is based on a household’s experience of accessing food and represents three aspects of food insecurity found to be universal across cultures: feelings of uncertainty or anxiety about household food supplies, perceptions that household food is of insufficient quality, and insufficient food intake [20, 30–32]. This scale consists of nine questions that ask the participant about their experience of food insecurity, with follow-up frequency questions. Responses to these questions are scored so that “never” receives a score of 0, “rarely” is scored 1, “sometimes” scored 2, and “often” scored 3, so that when summed, the lowest possible score is 0 and the highest is 27. Food insecurity is indicated by a higher score, with continuous scores typically divided into four categories, representing food-secure and mildly, moderately and severely food-insecure households according to the scheme recommended by the HFIAS Indicator Guide [30]. Not all studies included a result of the proportion of study participants who were food insecure before and/or after the intervention, rather reporting a mean [24, 26, 27], those that did use the HFIAS to measure food insecurity reported a range of food insecurity. For example, after a cash transfer, intervention close to 20% of participants was food insecure while around 35% of the control group were food insecure, with an increase in the percentage of houses who were food secure of 4–7% [22]. While Frongillo and Nguyen [29•] demonstrated an increase in food security from 55 to 75% for recently delivered women and 55 to 80% for pregnant women enrolled in a nutrition-focused antenatal education programme.

The next most common method to measure food insecurity was the Household Food Security Survey Module (HFSSM) (*n* = 3 studies). This survey was created by the US Department of Agriculture and can be administered as an 18-, 6-, or 4-item survey. This tool was developed to measure whether households have enough food or money to meet basic food needs, and what their behavioural and subjective responses to that condition were [33]. The full HFSSM consists of a set of 18 items, 8 of which are specific to households with children. It captures four types of household food insecurity experiences: uncertainty and worry, inadequate food quality, and insufficient food quantity for adults and children [34]. In accordance with the method proposed by Coleman-Jensen and Gregory [35], food security scores are combined to create one measure for level of food security for a household. A household is then defined as having high food security, marginal food insecurity, low food insecurity and very low food insecurity. Food security status is determined by the number of food insecure conditions and behaviours that the household reports. Studies that employed the HFSSM reported increased food security as a result of the intervention under study. For example, one study reported an increase in food security from 68.8% before an intervention that included food supplementation, increasing to 76.7% after the intervention [23], while another study that compared individual to group nutrition education saw an increase of 13 to 87% for group care and 9 to 78% for individual care [21].

One study used a more recently developed and less used survey to measure food insecurity. Frith, Naved [8] employed a questionnaire developed by Frongillo, Chowdhury [36]. This measure explores a range of strategies that people use to acquire food in addition to capturing food quality and quantity. Frith and Naved [8] use their description of food insecurity to understand other characteristics of their sample and do not report a percentage of those who are food secure.

## Discussion

This is the first systematic review to investigate interventions that seek to address food insecurity in pregnant women and new mothers. While the negative consequences of food insecurity are well-known, with some regions of the world disproportionally impacted by food insecurity, there remains limited evidence to inform best practice to target food insecurity among pregnant women and in the postnatal period. There is an urgent need to address food insecurity in this population group in the interest of best maternal and child health outcomes. The findings of this review suggest that while there are a range of possible interventions that seek to address food insecurity and hunger among pregnant and postpartum women, the limited number of robust evaluations or long-term interventions mean that evidence for any one intervention type is limited. Furthermore, the programmes and interventions that do exist are generally embedded within a single context or structure and as such may not be able to be widely implemented.

This review sought to explore interventions and programmes that had been published since 2000. Despite the 20-year time frame for the search, all the eleven studies that were identified through a systemic search had been published since 2010, with 6 of the 11 studies published since 2019. Given the recency of these publications, it may be that we are on the cusp of an increasing number of studies, evaluations, and reports of interventions that seek to address food insecurity in pregnant and postpartum women. With increasing attention being paid on the situation of food insecurity in both high- and low- and middle-income countries by governments and health bodies, it is possible that more researchers and interested parties will begin to create more structured and robust interventions to address food insecurity; however, a search of clinicaltrials.gov with the key term “food security” reveals only 70 clinical trials that are active, suggesting that if there is about to be an increase in responses to food insecurity interventions, they will continue to be context depended, of small scale, and therefore, possibly not published. A recent Australian scoping review of population level interventions that sought to address the socio-ecological determinants of food insecurity similarly found a lack of rigorous evaluation and coordination, suggesting that even in interventions aimed at addressing food insecurity in the general population, there is limited evidence surrounding what works [37].

Studies in this review were mostly conducted in low- and middle-income countries with the exclusion of the three that were conducted in the USA. Given the high prevalence of food insecurity in many low- and middle-income countries, it is not surprising that research has focused on these population groups [1]. However, this leaves a gap in the literature and the responses in high income countries, where some populations also experiences high rates of food insecurity [38]. Therefore, future research targeting “at risk” populations groups should be a public health priority globally, with increased efforts in designing culturally appropriate interventions across different antenatal settings being highly important. Interventions identified in this search reduced food insecurity through supplementation, in person group prenatal care, and unconditional or conditional cash transfers. Interventions included in this review were successful thanks to a range of aspects including early intervention, longer participation, and increased activity (both cash and food). Providing a range of actions was also found to be useful in improving food security, these included nutrition counselling, diet planning, weight gain monitoring, and micronutrient supplementation. Future interventions could draw on successful intervention components and adapt similar delivery methods and intervention lengths for targeted population groups. However, further work is required to carefully design potentially successful, sustainable, and cost-effective methods which can be embedded into existing antenatal healthcare systems.

Despite the clear benefits of interventions to address food insecurity, there remains a dearth in programmes that seek to address food insecurity during pregnancy. Potential reasons for the limited research in this area may be related to the known challenges with measuring food insecurity. While there are several standard measures of food insecurity as previously discussed, they often do not take other factors into account, including income and poverty, employment status, education level, location, ethnicity, and access to food and nutrition education programmes [39]. The challenges with both measuring food insecurity and identifying the main causes may deter some groups from intervening, and an issue further compounded by the fact that negative outcomes can occur months to years after the experience of food insecurity. In this current review, like that of Yii and Palermo [37], interventions with the biggest impact on reducing food insecurity were those that accounted for the influences of food insecurity.

The findings of this review are of importance to those working with pregnant and postpartum women, as it provides up to date evidence for the design of successful food insecurity alleviation interventions. It is recommended that a combination of resources is supplied to pregnant women and new mothers, to achieve a greater effect on reducing food insecurity. This includes a combination of prenatal care, together with nutrition counselling and education [21, 29•]. Useful delivery platforms which utilise technology-driven interventions might be further explored as they offer wide reach, are cost-effective, and alleviate clinician time pressures which have been identified as a barrier to health and nutrition management during pregnancy [40, 41]. Technology-assisted interventions involving text messaging and app-based delivery have shown to be promising and feasible in large populations and in the clinical, antenatal setting [42–44]. Furthermore, it is evident that longer participation in a programme determined a greater outcome [23]. This suggests that a longer programme, enabling earlier engagement, is more likely to have an impact on reducing food insecurity.

### Limitations

There are some limitations of this review that should also be acknowledged. While every attempt was made to ensure this review was comprehensive, additional articles may have been missed, particularly if articles were written in a language other than English. However, given this is the first review of its kind, with the inclusion of several databases and a range of broad key terms that include all dimensions of food security, the authors are confident that there is little information that is not presented here. Given the variety of approaches taken to measure food insecurity as found in this review, the various interventions under investigation, and the varying methods utilised, this review has not sought to present a meta-analysis. If in the future there can be some consistency in the use of measurement tools and approaches, a meta-analysis may be appropriate.

## Conclusion

This review emphasises the importance and urgency for the design and implementation of interventions that will address food insecurity among pregnant and postpartum women. Food insecurity is severe and common among pregnant women and new mothers, where adequate nutrition is vital during these life stages to avoid nutrient deficiencies within the mother and child, potentially impacting both short- and long-term health of the mother and child. Interventions and programmes that aim to reduce the impact of food insecurity during this period are needed but more importantly are those that are adequately described and evaluated so that knowledge, understandings, and facilitators can be shared across different regions and contexts.
